# Schizophrenia, Bipolar, or Major Depressive Disorder and Postacute Sequelae of COVID-19

**DOI:** 10.1001/jamanetworkopen.2025.40242

**Published:** 2025-10-29

**Authors:** Veer Vekaria, Rohith Kumar Thiruvalluru, Zoe Verzani, Sajjad Abedian, Mark Olfson, Braja Gopal Patra, Yunyu Xiao, Katherine S. Salamon, Karin Hoth, Frank Blancero, Maxwell M. Hornig-Rohan, Teresa Akintonwa, Mahfuza Sabiha, Mark G. Weiner, Thomas W. Carton, Rainu Kaushal, Jyotishman Pathak

**Affiliations:** 1 Weill Cornell Medicine, New York, New York; 2 New York State Psychiatric Institute, Columbia University, New York; 3 Nemours Children’s Health, Wilmington, Delaware; 4 Department of Psychiatry, University of Iowa, Iowa City; 5 RECOVER Patient, Caregiver, or Community Advocate Representative, New York, New York; 6 Louisiana Public Health Institute, New Orleans, Louisiana

## Abstract

**Importance:**

Given the increased vulnerability to COVID-19 among those with a serious mental illness (SMI), it remains unclear whether these individuals face a higher risk of developing postacute sequelae of SARS-CoV-2 (PASC). Understanding this association could inform secondary prevention efforts.

**Objective:**

To identify the risk of developing PASC in patients with an SMI.

**Design, Setting, and Participants:**

This longitudinal cohort study used data derived from large-scale electronic health records (EHRs) between March 2020 and April 2023, inclusive of 180-day follow-up. Patients included adults aged 21 years or older with a confirmed COVID-19 infection evidenced by a relevant laboratory result, diagnosis, or prescription order.

**Exposures:**

Evidence of an SMI diagnosis (schizophrenia, bipolar disorder, or recurrent major depressive disorder) recorded before COVID-19 infection.

**Main Outcomes and Measures:**

Evidence of PASC symptoms within 30 to 180 days’ follow-up after COVID-19 infection reported as odds ratios (OR) mutually adjusted for age, sex, race and ethnicity, insurance type, Charlson Comorbidity Index (CCI) score, and COVID-19 severity.

**Results:**

A total of 1 625 857 patients with a COVID-19 infection were included (mean [SD] age, 52 [17] years; 998 237 [61.4%] female, 204 237 [12.6%] non-Hispanic Black, 219 220 [13.5%] Hispanic, 833 411 [51.3%] non-Hispanic White, and 1 228 664 [75.6%] urban patients), of whom 258 523 (15.9%) had an SMI and 403 641 (24.8%) developed PASC. Individuals with an SMI had increased adjusted odds of developing PASC (OR, 1.10; 95% CI, 1.08-1.11; *P* < .001). Variables associated with greater odds of PASC among the study population included older age compared with age 22 to 34 years (35 to 44 years: OR, 1.04; 95% CI, 1.03-1.06; 45 to 64 years: OR, 1.11; 95% CI, 1.10-1.12; ≥65 years: OR, 1.18; 95% CI, 1.17-1.20), non-Hispanic Black and Hispanic compared with non-Hispanic White race and ethnicity (non-Hispanic Black: OR, 1.08; 95% CI, 1.07-1.10; Hispanic: OR, 1.12; 95% CI, 1.11-1.13), higher chronic disease burden vs no chronic disease (CCI 1 to 3: OR, 1.13; 95% CI, 1.12-1.14; CCI ≥4: OR, 1.23; 95% CI, 1.22-1.25), and hospitalization with initial COVID-19 infection vs no hospitalization (hospitalized: OR, 1.80; 95% CI, 1.77-1.82; hospitalized with ventilation: OR, 2.17; 95% CI, 2.12-2.22; *P* < .001). Compared with public insurance, commercial health insurance was associated with lower odds of PASC (OR, 0.85; 95% CI, 0.84-0.86).

**Conclusions and Relevance:**

In this cohort study of patients infected with COVID-19, patients with SMI compared with those without SMI were at increased risk of PASC, underscoring the need for coordinated mental health and COVID-19 care strategies.

## Key Points

**Question:**

Do adults with serious mental illnesses (schizophrenia, bipolar disorder, or recurrent major depressive disorder) have an increased risk of developing postacute sequelae of SARS-CoV-2 (PASC)?

**Findings:**

This cohort study of 1.6 million patients found that adults with serious mental illnesses had increased adjusted odds of developing PASC. Older individuals and those with non-Hispanic Black or Hispanic race and ethnicity, public health insurance, higher chronic disease burden, and greater severity of the initial COVID-19 infection had higher odds of PASC.

**Meaning:**

These results suggest the need for coordinated approaches that simultaneously treat and seek to prevent PASC among adults with serious mental illnesses.

## Teaser

This cohort study evaluates the risk of developing postacute sequelae of COVID-19 in patients with schizophrenia, bipolar, or major depressive disorder.

## Introduction

The COVID-19 pandemic has had a profound impact on public health, with over 111 million cases and more than 1.2 million deaths in the US alone by mid-2024.^1^ The mortality associated with COVID-19 has underscored the severity of the viral infections and the ongoing need for research into its long-term effects.

Current research summarizes these long-term effects as postacute sequelae of SARS-CoV-2 infection (PASC). PASC is defined as ongoing, relapsing, or new symptoms or other health effects occurring after the acute phase of SARS-CoV-2 infection (ie, that present ≥4 weeks after the acute infection).^2^ Approximately 20% of patients who recover from the initial infection experience persistent symptoms or develop new medical conditions, including dyspnea, fatigue, cognitive dysfunction, anxiety, and coagulation disorders.^3,4^ These symptoms can last for months, impacting quality of life and requiring medical attention that persists beyond the acute infection phase.^5^

Individuals with serious mental illness (SMI) have an increased risk of COVID-19 infection and COVID-19–related mortality.^6^ SMI—defined by the American Psychological Association as a diagnostic group of psychiatric disorders including schizophrenia, bipolar disorder, and recurrent major depressive disorder (MDD)—impacts 5% of US adults.^7^ The increased COVID-19 infection and mortality risks of adults with SMI are due in part to limited general medical care access, treatment adherence challenges, and the presence of comorbidities such as cardiovascular disease and diabetes.^8^ Moreover, pandemic-induced stressors, such as anxiety, social isolation, and abrupt changes in daily routines, may have further complicated the clinical course and medical care of adults with SMI.^9^

Beyond the increased vulnerability of individuals with an SMI to COVID-19, the relationship between SMI and development of PASC requires further investigation. Specifically, it is unclear whether following COVID-19 infection, patients with SMI compared with those without SMI are at increased risk of developing long-term COVID-19 sequelae. An understanding of this association would have implications for secondary prevention of PASC. Using data derived from large-scale, routinely collected electronic health records (EHRs), this study aims to examine the association between prior SMI and the subsequent development of PASC in clinical practice patients infected by COVID-19. We hypothesized that, conditioned on having a COVID-19 infection, SMI would be associated with increased odds of PASC, given that SMI is associated with greater severity of COVID-19 infections and the latter is associated with increased risk PASC.

## Methods

### Data Source

A longitudinal cohort study was conducted using data derived from March 2020 and April 2023 using deidentified electronic health records from the National Patient-Centered Clinical Research Network (PCORnet), which includes 28 diverse health care sites with a significant known adult population, capturing over 22 million patients.^10,11^ Institutional review board approval was obtained from the Biomedical Research Alliance of New York (BRANY). The protocol was reviewed in accordance with institutional guidelines, and BRANY waived the requirements for informed consent and HIPAA authorization because data were deidentified. The reporting of this study adhered to the Strengthening the Reporting of Observational Studies in Epidemiology (STROBE) reporting guideline.^12^

### Study Population

eTable 1 in Supplement 1 shows the patient attrition associated with each of the inclusion and exclusion filtering criteria used to derive the final study cohort with an eligible COVID-19 infection. This study was restricted to adults with a documented COVID-19 infection between March 2020 and October 2022 (ie, 6 months before the study end date, to ensure sufficient time for PASC to develop). Supporting evidence of a COVID-19 infection included a positive polymerase chain reaction or antigen test, an *International Statistical Classification of Diseases and Related Health Problems, Tenth Revision (ICD-10)*–coded diagnosis (U07.1), or a prescription order for nirmatrelvir or ritonavir (Paxlovid) or remdesivir (Veklury). To increase the likelihood of continuous enrollment and mitigate loss to follow-up, patients were required to have at least 1 encounter 1 year before the COVID-19 infection, and at least 1 follow-up visit 30 or more days after. The first such recorded evidence of COVID-19 during the observation period where the patient was also aged 21 years or older established the index event. Patients with missing or unknown sex were excluded.

### Exposures

Among included patients with a COVID-19 infection, those with prior SMI were identified as those with 2 or more outpatient encounters or 1 or more psychiatric hospitalizations in their entire available EHR record preceding the index COVID-19 infection where the encounter diagnosis code (*ICD-10*) included schizophrenia (F20, F25, F60), bipolar disorder (F31), or recurrent major depressive disorder (MDD) (F33). For the purposes of this study, SMI was assumed to be a persistent, chronic condition within the study period, and SMI diagnoses recorded after the index date in patients with prior SMI were considered consistent with this chronicity.^7^ Those with PASC were identified using the code set and procedures described in eTable 2 in Supplement 1. Subtypes of PASC, categorized by affected system, included blood-related, circulatory-related, digestive-related, endocrine-related, musculoskeletal-related, neurological-related, respiratory-related, and skin-related postacute sequelae.

### Measures and Outcomes

Patients with COVID-19 were characterized by sociodemographic characteristics (including age, sex, race and ethnicity [Hispanic; non-Hispanic Asian, Black, White, or other; and missing or unknown], insurance type, rurality, and overall social vulnerability index [SVI]) and clinical characteristics (Charlson Comorbidity Index [CCI] score, past-year health care services utilization, COVID-19-related variant, severity, treatment, and mortality), all measured based on the index COVID-19 infection date. Results were stratified by patients with PASC compared with patients without PASC.^13^ Race and ethnicity classifications were based on patient self-report from the EHR, and assessed in this study as a proxy for exposure to health disparities. Insurance type was ascertained as the most recent nonnull insurance documented on or before the index COVID-19 infection date, and aggregated as commercial or public following physician review. For rurality and SVI, crosswalk files from the US Department of Housing and Urban Development were used to map patients’ most recently recorded zip code and census tract information to the county level. The zip code-level information was then mapped to 2010 rural-urban commuting area codes from the US Department of Agriculture, and county-level information was mapped to 2022 SVI scores from the US Centers for Disease Control and Prevention (CDC).^14^ CCI scores were computed up to and including the index date; a CCI score of 0 indicated available EHR history without CCI-qualifying conditions. Past-year health care services utilization (mental health, outpatient, inpatient, emergency department [ED], and telehealth visits) were measured relative to the index date. The COVID-19 variant was established based on the dominant strain in the US on the index date (see eTable 3 in Supplement 1).^15,16^ Finally, COVID-19 severity was ascertained at 4 mutually exclusive levels: hospitalized for COVID-19 with ventilation (most severe), hospitalized for COVID-19, incidental hospitalization (ie, admitted for unrelated indications with an incidentally positive test), or not hospitalized (least severe).^17^

### Statistical Analysis

Descriptive statistics (including mean [SD], frequency, and percentage) were calculated to summarize the demographic and clinical profile of the study cohorts with COVID-19. To compare sociodemographic and clinical characteristics between patients with vs without PASC, the Welch 2-sample *t* test was used for continuous variables and Pearson χ^2^ test was used for categorical variables (Table 1). A multivariable logistic regression model adjusted for age, sex, race and ethnicity, insurance type, and CCI score, and COVID-19 severity was fitted to evaluate the association between the exposure (prior SMI) and the outcome (PASC). Statistical significance was established using a 2-sided significance level of .05. The data met the respective assumptions of all statistical tests performed. R version 4.4.0 (R Project for Statistical Computing) was used for all analyses.^18^

**Table 1.** Sociodemographic and Clinical Characteristics of Patients in the Overall COVID-19 Cohort and Stratified by Patients With vs Without Postacute Sequelae of COVID-19 (PASC)

Characteristic	Total, No. (%) (N = 1 625 857)	PASC, No. (%) (n = 403 641)	No PASC, No. (%) (n = 1 222 216)
Serious mental illness	258 523 (15.9)	71 562 (17.7)	186 961 (15.3)
Schizophrenia	11 201 (0.7)	3321 (0.8)	7880 (0.6)
Bipolar disorder	35 469 (2.2)	10 243 (2.5)	25 226 (2.1)
Recurrent MDD	239 747 (14.7)	65 771 (16.3)	173 976 (14.2)
Sociodemographic characteristics			
Age at infection, y			
Mean (SD)	52 (17)	54 (18)	51 (17)
22 to 34	331 119 (20.4)	72 257 (17.9)	258 862 (21.2)
35 to 44	288 848 (17.8)	65 906 (16.3)	222 942 (18.2)
45 to 64	582 648 (35.8)	144 378 (35.8)	438 270 (35.9)
≥65	423 242 (26.0)	121 100 (30.0)	302 142 (24.7)
Sex			
Female	998 237 (61.4)	254 320 (63.0)	743 917 (60.9)
Male	627 620 (38.6)	149 321 (37.0)	478 299 (39.1)
Race and ethnicity			
Hispanic	219 220 (13.5)	56 270 (13.9)	162 950 (13.3)
Non-Hispanic Asian	50 400 (3.1)	11 454 (2.8)	38 946 (3.2)
Non-Hispanic Black	204 237 (12.6)	53 086 (13.2)	151 151 (12.4)
Non-Hispanic White	833 411 (51.3)	197 434 (48.9)	635 977 (52.0)
Non-Hispanic other	65 009 (4.0)	16 314 (4.0)	48 695 (4.0)
Missing or unknown	253 580 (15.6)	69 083 (17.1)	184 497 (15.1)
Insurance type			
Public	362 016 (22.3)	102 671 (25.4)	259 345 (21.2)
Commercial	717 942 (44.2)	163 339 (40.5)	554 603 (45.4)
Unknown or other	545 899 (33.6)	137 631 (34.1)	408 268 (33.4)
Rurality			
Urban	1 228 664 (75.6)	300 646 (74.5)	928 018 (75.9)
Large town	101 050 (6.2)	25 965 (6.4)	75 085 (6.1)
Rural	21 808 (1.3)	5581 (1.4)	16 227 (1.3)
Small town	35 725 (2.2)	8845 (2.2)	26 880 (2.2)
Missing	238 610 (14.7)	62 604 (15.5)	176 006 (14.4)
Social vulnerability index			
Mean (SD)	0.55 (0.26)	0.57 (0.26)	0.55 (0.26)
Quartile 1 (least vulnerable)	143 244 (8.8)	32 065 (7.9)	111 179 (9.1)
Quartile 2	499 480 (30.7)	115 558 (28.6)	383 922 (31.4)
Quartile 3	344 580 (21.2)	89 731 (22.2)	254 849 (20.9)
Quartile 4 (most vulnerable)	399 763 (24.6)	103 630 (25.7)	296 133 (24.2)
Unknown	238 790 (14.7)	62 657 (15.5)	176 133 (14.4)
Clinical characteristics			
CCI score			
0	813 575 (50.0)	181 847 (45.1)	631 728 (51.7)
1 to 3	565 258 (34.8)	147 955 (36.7)	417 303 (34.1)
≥4	229 069 (14.1)	70 674 (17.5)	158 395 (13.0)
Unknown	17 955 (1.1)	3165 (0.8)	14 790 (1.2)
Past year health care services utilization			
Mental health visit	457 601 (28.1)	134 252 (33.3)	323 349 (26.5)
Emergency department visit	100 687 (6.2)	32 195 (8.0)	68 492 (5.6)
Inpatient visit	73 028 (4.5)	25 534 (6.3)	47 494 (3.9)
Outpatient visit	401 356 (24.7)	117 158 (29.0)	284 198 (23.3)
Telehealth visit	115 851 (7.1)	34 138 (8.5)	81 713 (6.7)
COVID-19 variant			
Alpha	177 473 (10.9)	44 685 (11.1)	132 788 (10.9)
Ancestral	376 813 (23.2)	89 357 (22.1)	287 456 (23.5)
Delta	187 331 (11.5)	46 847 (11.6)	140 484 (11.5)
Omicron BA.1.1 - BA.2	380 862 (23.4)	92 343 (22.9)	288 519 (23.6)
Omicron BA.2.12.1 - BA.5	386 495 (23.8)	97 979 (24.3)	288 516 (23.6)
Omicron BQ.1.1 - XBB.1.5	116 883 (7.2)	32 430 (8.0)	84 453 (6.9)
COVID-19 severity			
Not hospitalized (least severe)	1 463 710 (90.0)	341 570 (84.6)	1 122 140 (91.8)
Incidental hospitalization	11 587 (0.7)	3951 (1.0)	7636 (0.6)
Hospitalized	114 873 (7.1)	43 373 (10.7)	71 500 (5.9)
Hospitalized with ventilation (most severe)	35 687 (2.2)	14 747 (3.7)	20 940 (1.7)
COVID-19 treatment			
Nirmatrelvir or ritonavir order	171 594 (10.6)	41 095 (10.2)	130 499 (10.7)
Remdesivir order	23 356 (1.4)	9623 (2.4)	13 733 (1.1)
COVID-19 death	30 896 (1.9)	15 150 (3.8)	15 746 (1.3)
PASC subtype			
Blood-related	NA	31 703 (7.9)	NA
Circulatory-related	NA	77 163 (19.1)	NA
Digestive-related	NA	74 039 (18.3)	NA
Endocrine-related	NA	76 476 (18.9)	NA
Musculoskeletal-related	NA	54 477 (13.5)	NA
Neurological-related	NA	133 203 (33.0)	NA
Respiratory-related	NA	107 434 (26.6)	NA
Skin-related	NA	16 932 (4.2)	NA

Abbreviations: CCI, Charlson comorbidity index; MDD, major depressive disorder; NA, not applicable.

## Results

Table 1 shows the distribution of patients with COVID-19 across various demographic and clinical variables. A total of 1 625 827 patients with a COVID-19 infection were included in this analysis (998 237 [61.4%] female; 219 220 [13.5%] Hispanic; 50 400 [3.1%] non-Hispanic Asian; 204 237 [12.6%] non-Hispanic Black; 833 411 [51.3%] non-Hispanic White; and 65 009 [4.0%] non-Hispanic other race patients), of whom 403 641 (24.8%) developed PASC and 258 523 (15.9%) had a prior SMI diagnosis, including schizophrenia (11 201 patients [0.7%]), bipolar disorder (35 469 patients [2.2%]), and recurrent MDD (239 747 patients [14.7%]). Patients in the overall sample with COVID-19 had a mean (SD) age of 52 (17) years, and the largest age group represented was patients aged 45 to 64 years (582 648 patients [35.8%]) Among patients with a prior SMI, 71 562 (27.7%) developed PASC (see eTable 4 in Supplement 1); conversely, among those who developed PASC, 71 562 (17.7%) had a prior SMI, including recurrent MDD (65 771 patients [16.3%]), bipolar disorder (10 243 patients [2.5%]), and schizophrenia (3321 patients [0.8%]). More patients with COVID-19 had commercial vs public health insurance (717 942 patients [44.2%] vs 362 016 patients [22.3%], respectively) and lived in urban vs rural areas (1 228 664 patients [75.6%] vs 21 808 patients [1.3%], respectively). On a scale of 0 (least vulnerable) to 1 (most vulnerable), the overall social vulnerability of the COVID-19 sample was 0.55. Additional results showing the distribution of patients across individual social vulnerability indicators (socioeconomic status, household characteristics, and housing type and transportation) are shown in eTable 5 in Supplement 1.

In terms of clinical variables, half of identified individuals had a CCI score of 0 (813 575 patients [50.0%]), with progressively fewer patients having CCI scores between 1 and 3 (565 258 patients [34.8%]) or 4 or more (229 069 patients [14.1%]). A larger proportion of patients with an SMI had a past-year mental health visit (199 972 patients [77.4%]) compared with those without SMI (257 629 patients [18.8%]) (see eTable 4 in Supplement 1). A roughly even distribution of patients was observed across COVID-19 variants, including the ancestral (376 813 patients [23.2%]), omicron BA.2 (380 862 patients [23.4%]), and omicron BA.5 (386 495 patients [23.8%]) variants. Most patients had relatively low COVID-19 severity (ie, not hospitalized; 1 463 710 patients [90.0%]), and less than 10% of patients were hospitalized (114 873 patients [7.1%]) or hospitalized with ventilation (36 687 patients [2.2%]). Treatments for COVID-19 included prescription orders of nirmatrelvir or ritonavir (171 594 patients [10.6%]) and remdesivir (23 356 patients [1.4%]). Death resulting from COVID-19 accounted for 30 896 individuals (1.9%) in the study sample. Among patients with PASC, the most common subtypes were neurological-related (133 203 patients [33.0%]), respiratory-related (107 434patients [26.6%]), circulatory-related (77 163 patients [19.1%]), and endocrine-related (76 476 patients [18.9%]).

Table 2 summarizes the association between prior SMI and developing PASC, based on the results of a multivariable logistic regression model adjusted for age, sex, race and ethnicity, insurance type, CCI score, and COVID-19 severity. The adjusted results suggest that patients with an SMI had increased odds of developing PASC compared with patients without an SMI (OR, 1.10; 95% CI, 1.08-1.11; *P* < .001), with this estimate reflecting the association for the reference profile (aged 22 to 34 years, female sex, non-Hispanic White race and ethnicity, public insurance, CCI score 0, and not hospitalized for COVID-19). Additionally, separate logistic regression models fitted for patients with SMI (eTable 6 in Supplement 1) and without SMI (eTable 7 in Supplement 1) provide further insight into how associations between covariates and PASC may differ by SMI.

**Table 2.** Logistic Regression Results Summarizing the Association Between Serious Mental Illness and Postacute Sequelae of COVID-19 (PASC)

Variable	Unadjusted		Mutually adjusted	
	OR (95% CI)	*P* value	OR (95% CI)	*P* value
Serious mental illness	1.19 (1.18-1.20)	<.001	1.10 (1.08-1.11)	<.001
Age at infection, y				
22 to 34	1 [Reference]	NA	1 [Reference]	NA
35 to 44	1.06 (1.05-1.07)	<.001	1.04 (1.03-1.06)	<.001
45 to 64	1.18 (1.17-1.19)	<.001	1.11 (1.10-1.12)	<.001
≥65	1.44 (1.42-1.45)	<.001	1.18 (1.17-1.20)	<.001
Sex				
Female	1 [Reference]	NA	1 [Reference]	NA
Male	0.91 (0.91-0.92)	<.001	0.88 (0.87-0.89)	<.001
Race and ethnicity				
Hispanic	1.11 (1.10-1.12)	<.001	1.12 (1.11-1.13)	<.001
Non-Hispanic Asian	0.95 (0.93-0.97)	<.001	0.98 (0.96-1.00)	.07
Non-Hispanic Black	1.13 (1.12-1.14)	<.001	1.08 (1.07-1.10)	<.001
Non-Hispanic White	1 [Reference]	NA	1 [Reference]	NA
Non-Hispanic other	1.08 (1.06-1.10)	<.001	1.07 (1.05-1.09)	<.001
Missing or unknown	1.21 (1.19-1.22)	<.001	1.22 (1.21-1.23)	<.001
Insurance type				
Public	1 [Reference]	NA	1 [Reference]	NA
Commercial	0.74 (0.74-0.75)	<.001	0.85 (0.84-0.86)	<.001
Unknown or other	0.85 (0.84-0.86)	<.001	0.91 (0.90-0.92)	<.001
CCI score				
0	1 [Reference]	NA	1 [Reference]	NA
1 to 3	1.23 (1.22-1.24)	<.001	1.13 (1.12-1.14)	<.001
≥4	1.55 (1.53-1.57)	<.001	1.23 (1.22-1.25)	<.001
Unknown	0.74 (0.72-0.77)	<.001	0.72 (0.69-0.75)	<.001
COVID-19 severity				
Not hospitalized (least severe)	1 [Reference]	NA	1 [Reference]	NA
Incidental hospitalization	1.70 (1.64-1.77)	<.001	1.59 (1.53-1.65)	<.001
Hospitalized	1.99 (1.97-2.02)	<.001	1.80 (1.77-1.82)	<.001
Hospitalized with ventilation (most severe)	2.31 (2.26-2.36)	<.001	2.17 (2.12-2.22)	<.001

Abbreviations: CCI, Charlson comorbidity index; NA, not applicable.

Relative to patients in the youngest age group (22 to 34 years), patients in the 35 to 44 years (OR, 1.04; 95% CI, 1.03-1.06), 45 to 64 years (OR, 1.11; 95% CI, 1.10-1.12), and 65 years or older (OR, 1.18; 95% CI, 1.17-1.20) age groups were more likely to develop PASC (*P* < .001). Male patients were less likely than female patients to develop PASC (OR, 0.88; 95% CI, 0.87-0.89; *P* < .001). Compared with patients who identified as non-Hispanic White, those who identified as Hispanic (OR, 1.12; 95% CI, 1.11-1.13) or non-Hispanic Black (OR, 1.08; 95% CI, 1.07-1.10) were more likely to develop PASC (*P* < .001). Additionally, patients with commercial health insurance plans were less likely than those who were publicly insured to develop PASC (OR, 0.85; 95% CI, 0.84-0.86; *P* < .001). Those with CCI scores between 1 and 3 (OR, 1.13; 95% CI, 1.12-1.14) and 4 or more (OR, 1.23; 95% CI, 1.22-1.25) were more likely than those with a CCI score of 0 to develop PASC (*P* < .001). Lastly, increased COVID-19 severity, including those who were hospitalized for COVID-19 (OR, 1.80; 95% CI, 1.77-1.82) and in particular those hospitalized with ventilation (OR, 2.17; 95% CI, 2.12-2.22), was associated with increased odds of developing PASC (*P* < .001).

Independently, each of the individual prior SMI categories—schizophrenia (OR, 1.07; 95% CI, 1.02-1.11; *P* = .002), bipolar disorder (OR, 1.14; 95% CI, 1.12-1.17; *P* < .001), and recurrent MDD (OR, 1.08; 95% CI, 1.07-1.09; *P* < .001)—were associated with increased odds of developing PASC, and the directional associations for each variable as described for any prior SMI were also sustained for each prior SMI category (see eTable 8 for schizophrenia, eTable 9 for bipolar disorder, and eTable 10 for recurrent MDD in Supplement 1). These results were also consistent with the results of an augmented logistic regression adding in adjustments for social vulnerability indicators (socioeconomic status, household characteristics, and housing type and transportation), which are presented in eTable 11 in Supplement 1.

## Discussion

Beyond previously described vulnerabilities of patients with SMI to COVID-19 infections and mortality, the present findings support their increased vulnerability to development of PASC following COVID-19 infection. This analysis also highlights differential risks associated with factors such as age, sex, race and ethnicity, insurance status, COVID-19-severity, and other sociodemographic determinants, suggesting that these variables influence health outcomes in this population. Finally, the consistency of these findings in each of the SMI categories—schizophrenia, bipolar disorder, and recurrent MDD—suggests transdiagnostic vulnerabilities to long-term adverse effects of COVID-19 among adults with severe mental illnesses.

The finding that 27.7% of patients with SMI developed PASC, with significantly increased adjusted odds (OR = 1.10), aligns with research indicating that PASC commonly occurs among adults with SMI.^3^ Chronic stress and immune dysregulation associated with an SMI can exacerbate the risk of PASC, making these individuals more prone to persistent symptoms due to underlying physical and mental health vulnerabilities.^19^ The heterogeneous distribution of PASC subtypes suggests overlapping symptoms affecting multiple systems with diverse underlying pathologies.^20^ The impact of recurrent MDD (14.7%) is relevant since depression is associated with increased COVID-19 risk and poorer recovery.^21^ Although recurrent MDD was the dominant SMI diagnosis, the distribution of SMI subtypes mirrored CDC estimates, with MDD generally more prevalent than other SMIs.^22^ Furthermore, the directional findings were consistent in logistic regressions run separately for each SMI subtype, suggesting that these findings are representative of SMI overall and not recurrent MDD alone. Future directions may explore PASC risk in patients with other neuropsychiatric disorders (beyond schizophrenia, bipolar disorder, and recurrent MDD) and identify clinical characteristics associated with increased risk.

Also, the finding that most study participants who developed PASC did not require hospitalization for COVID-19 supports other studies showing that PASC can occur even after mild or moderate cases of the viral infection.^23^ For patients with an SMI, even less severe acute infections can significantly impact long-term health due to increased levels of physiological and psychological stress.^24^ Additionally, the relatively high proportion of patients with commercial insurance (44.2%), including among those who developed PASC or had a prior SMI, raises the possibility that better access to health care services does not eliminate the risk of PASC following COVID-19 infection.^25,26^

The largest age group was 45 to 64 years, a range known to have increased risks of severe COVID-19 and PASC.^27,28^ This age group is more likely to harbor accumulated physical and mental health issues, including unrecognized or untreated mental illnesses, which can increase the risk of long-term complications including PASC.^29,30^ Older adults with an SMI are particularly vulnerable due to age-related decline and stress associated with managing other chronic health conditions.^31^

Furthermore, Hispanic and Black patients were more likely to develop PASC compared with White patients, which is important given that race can serve as a proxy for health disparities. These disparities are important in the context of SMI and PASC, because racially and ethnically minoritized individuals often face systemic barriers in health care that can delay diagnosis and management of both conditions.^32,33^ The stress, discrimination, and adverse social determinants of health (SDOH) experienced by these groups can further increase the risk of PASC, especially among those with an SMI.^34^ Given these increased vulnerabilities, health systems may respond by expanding screening for PASC and associated risk factors in Black and Hispanic communities, supporting training on implicit bias among mental health practitioners, and addressing relevant SDOH such as housing instability and social support among patients with SMI.^35^

Patients with multiple comorbidities (measured by CCI) also had increased odds of developing PASC. Co-occurring physical health issues complicate recovery from COVID-19, as the burden of these conditions can strain the body’s resources and hinder its ability to combat long-term effects of the virus.^36,37^ Similarly, the severity of the initial COVID-19 infection remains a strong predictor of developing PASC, with more severe cases leading to higher odds of long-term complications.^38,39^ This is concerning for patients with COVID-19 with an SMI, of whom a larger proportion were hospitalized compared with those without an SMI.^19^ The stress and damage from severe COVID-19, coupled with recovery challenges inherent to SMI, amplify the risk of PASC.^19^

### Limitations

Several limitations should be noted. First, the PCORnet EHR dataset includes only patients who had encounters with affiliated health systems, excluding those who did not seek care or accessed care elsewhere. Patients who self-diagnosed COVID-19 using in-home tests and self-managed symptoms without clinical documentation would have been misclassified as COVID-negative and excluded. Requiring a follow-up visit 30 or more days postinfection may also limit generalizability by omitting those who died shortly after infection. Second, many health systems restricted elective visits and hospitalizations during the pandemic, which may have reduced postinfection outcome data. Third, prescription data capture orders only, not filled or dispensed medications, so results may not necessarily reflect treatment adherence. Fourth, symptom overlap between depression and PASC (eg, fatigue or cognitive issues) may lead to misclassification and partially explain the increased PASC odds observed in recurrent MDD.^40^ Fifth, urban patients are overrepresented because participating sites are primarily in urban areas.^41^ Additionally, given the relatively large sample size, some statistically significant results may overstate their practical impact and should be interpreted alongside effect sizes to better contextualize their magnitude.^42^

## Conclusions

In this large multicenter national cohort study of patients with confirmed COVID-19 infections, patients with an SMI were significantly more vulnerable to PASC. Our findings suggest that various sociodemographic factors, such as age, race and ethnicity, and insurance status, are associated with PASC outcomes. The consistent patterns observed across different types of SMI support a need for targeted interventions to address the long-term effects of COVID-19. The increased prevalence of comorbidities and chronic stress in individuals with an SMI might contribute to their higher risk of developing PASC, especially given the overlapping symptoms with conditions like recurrent MDD, though this requires further study. These results emphasize the need for coordinated approaches that simultaneously treat and seek to prevent PASC among adults with SMI.

## Notes

Supplement 1. eTable 1. Inclusion and Exclusion Criteria Used to Select the COVID-19 Study Cohort eTable 2. PASC Code Set and Procedure eTable 3. Dominant COVID-19 Variant in the US at Different Date Ranges eTable 4. Sociodemographic and Clinical Characteristics of Patients in the Overall COVID-19 Cohort and Stratified by Patients With vs Without a Prior SMI eTable 5. Distribution of Patients With Social Vulnerability Indicators (First Quartile = Least Vulnerable, Fourth Quartile = Most Vulnerable) eTable 6. Logistic Regression Results Summarizing the Association Between Covariates and PASC Among Patients With SMI Only eTable 7. Logistic Regression Results Summarizing the Association Between Covariates and PASC Among Patients Without SMI Only eTable 8. Logistic Regression Results Summarizing the Association Between Prior Schizophrenia and PASC eTable 9. Logistic Regression Results Summarizing the Association Between Prior Bipolar Disorder and PASC eTable 10. Logistic Regression Results Summarizing the Association Between Prior Recurrent MDD and PASC eTable 11. Logistic Regression Summary of Predictors of Developing PASC Including Adjustments for Social Vulnerability Indicators (Socioeconomic Status, Household Characteristics, Housing Type and Transportation)

Supplement 2. Nonauthor Collaborators

Supplement 3. Data Sharing Statement
