# Supplementary material for: Schizophrenia, Bipolar, or Major Depressive Disorder and Postacute Sequelae of COVID-19
Source: JAMA Netw Open. 2025 Oct 29;8(10):e2540242. doi: 10.1001/jamanetworkopen.2025.40242 (PMC12573031; doi:10.1001/jamanetworkopen.2025.40242)
Supplement: Supplement 1. — eTable 1. Inclusion and Exclusion Criteria Used to Select the COVID-19 Study Cohort eTable 2. PASC Code Set and Procedure eTable 3. Dominant COVID-19 Variant in the US at Different Date Ranges eTable 4. Sociodemographic and Clinical Characteristics of Patients in the Overall COVID-19 Cohort and Stratified by Patients With vs Without a Prior SMI eTable 5. Distribution of Patients With Social Vulnerability Indicators (First Quartile = Least Vulnerable, Fourth Quartile = Most Vulnerable) eTable 6. Logistic Regression Results Summarizing the Association Between Covariates and PASC Among Patients With SMI Only eTable 7. Logistic Regression Results Summarizing the Association Between Covariates and PASC Among Patients Without SMI Only eTable 8. Logistic Regression Results Summarizing the Association Between Prior Schizophrenia and PASC eTable 9. Logistic Regression Results Summarizing the Association Between Prior Bipolar Disorder and PASC eTable 10. Logistic Regression Results Summarizing the Association Between Prior Recurrent MDD and PASC eTable 11. Logistic Regression Summary of Predictors of Developing PASC Including Adjustments for Social Vulnerability Indicators (Socioeconomic Status, Household Characteristics, Housing Type and Transportation) [file jamanetwopen-e2540242-s001.pdf]

Supplemental Online Content

Vekaria V, Thiruvalluru RK, Verzani Z, et al. Association of schizophrenia, bipolar, or major depressive disorder with PASC in patients with COVID-19. *JAMA Netw Open*. 2025;8(10):e2540242. doi:10.1001/jamanetworkopen.2025.40242

eTable 1. Inclusion and Exclusion Criteria Used to Select the COVID-19 Study Cohort
eTable 2. PASC Code Set and Procedure
eTable 3. Dominant COVID-19 Variant in the US at Different Date Ranges
eTable 4. Sociodemographic and Clinical Characteristics of Patients in the Overall COVID-19 Cohort and Stratified by Patients With vs Without a Prior SMI
eTable 5. Distribution of Patients With Social Vulnerability Indicators (First Quartile = Least Vulnerable, Fourth Quartile = Most Vulnerable)
eTable 6. Logistic Regression Results Summarizing the Association Between Covariates and PASC Among Patients With SMI Only
eTable 7. Logistic Regression Results Summarizing the Association Between Covariates and PASC Among Patients Without SMI Only
eTable 8. Logistic Regression Results Summarizing the Association Between Prior Schizophrenia and PASC
eTable 9. Logistic Regression Results Summarizing the Association Between Prior Bipolar Disorder and PASC
eTable 10. Logistic Regression Results Summarizing the Association Between Prior Recurrent MDD and PASC
eTable 11. Logistic Regression Summary of Predictors of Developing PASC Including Adjustments for Social Vulnerability Indicators (Socioeconomic Status, Household Characteristics, Housing Type and Transportation)

This supplemental material has been provided by the authors to give readers additional information about their work.

© 2025 Vekaria V et al. *JAMA Network Open*.

eTable 1. Inclusion and Exclusion Criteria Used to Select the COVID-19 Study Cohort

**Criteria**	**Patients**
Patients with available EHR data (March 2020 to April 2023)	23,977,586
Patients with an index COVID-19 infection (March 2020 to October 2022)	3,071,281
Patients with any encounter within 1 year before index event	2,469,921
Patients with any encounter within ≥30 days after index event	2,054,707
Patients aged ≥21 years on index date	1,650,157
Patients without missing or unknown sex	1,649,910
Final COVID-19 study cohort	1,625,857

eTable 2. PASC Code Set and Procedure

**Item**	**Details**
PASC code set	PASC code set (link to GitHub)
Procedure	We used ICD codes under categories outlined in the code set to identify diagnoses that could be accounted for PASC. We followed the steps below to identify whether the diagnosis of interest is a PASC diagnosis or a comorbidity.<br><br>First, we determined each patient's index date and defined a blackout period by excluding all recorded diagnoses from 7 days prior to 30 days after the index date. Next, we identified the earliest instance of a recorded diagnosis category within 180 days after the index event. A diagnosis was classified as a comorbidity if its earliest instance occurred before the index date; if it occurred after the index date, it was classified as a PASC diagnosis. A patient's overall PASC status was determined by identifying those with at least one PASC diagnosis in their records. Additionally, the patient's PASC subphenotype was defined by mapping the identified PASC diagnoses to the affected organ system.

eTable 3. Dominant COVID-19 Variant in the US at Different Date Ranges

**Date range**	**Dominant variant**
3/1/2020 – 12/31/2020	Ancestral
1/1/2021 – 5/31/2021	Alpha
6/1/2021 – 11/30/2021	Delta
12/1/21 – 4/30/2022	Omicron BA.1.1 - BA.2
5/1/2022 – 11/30/2022	Omicron BA.2.12.1 - BA.5
12/1/2022 – Present	Omicron BQ.1.1 - XBB.1.5

© 2025 Vekaria V et al. *JAMA Network Open*.

eTable 4. Sociodemographic and Clinical Characteristics of Patients in the Overall COVID-19 Cohort and Stratified by Patients With vs Without a Prior SMI

Characteristic	Total, No. (%)	SMI, No. (%)	No SMI, No. (%)
Total	1,625,857	258,523	1,367,334
**Post-acute Sequelae of SARS-CoV-2 (PASC)**	403,641 (24.8)	71,562 (27.7)	332,079 (24.3)
**Sociodemographic characteristics**			
**Age at infection, y**			
Mean (SD)	52 (17)	52 (17)	52 (17)
22 to 34	331,119 (20.4)	47,666 (18.4)	283,453 (20.7)
35 to 44	288,848 (17.8)	44,844 (17.3)	244,004 (17.8)
45 to 64	582,648 (35.8)	96,677 (37.4)	485,971 (35.5)
≥65	423,242 (26.0)	69,336 (26.8)	353,906 (25.9)
**Sex**			
Female	998,237 (61.4)	185,589 (71.8)	812,648 (59.4)
Male	627,620 (38.6)	72,934 (28.2)	554,686 (40.6)
**Race and ethnicity**			
Hispanic	219,220 (13.5)	28,955 (11.2)	190,265 (13.9)
Non-Hispanic Asian	50,400 (3.1)	3,726 (1.4)	46,674 (3.4)
Non-Hispanic Black	204,237 (12.6)	31,600 (12.2)	172,637 (12.6)
Non-Hispanic White	833,411 (51.3)	153,406 (59.3)	680,005 (49.7)
Non-Hispanic other	65,009 (4.0)	7,205 (2.8)	57,804 (4.2)
Missing or unknown	253,580 (15.6)	33,631 (13.0)	219,949 (16.1)
**Insurance type**			
Public	362,016 (22.3)	79,889 (30.9)	282,127 (20.6)
Commercial	717,942 (44.2)	112,212 (43.4)	605,730 (44.3)
Unknown or other	545,899 (33.6)	66,422 (25.7)	479,477 (35.1)
**Rurality**			
Urban	1,228,664 (75.6)	197,603 (76.4)	1,031,061 (75.4)
Large town	101,050 (6.2)	17,345 (6.7)	83,705 (6.1)
Rural	21,808 (1.3)	3,645 (1.4)	18,163 (1.3)
Small town	35,725 (2.2)	7,007 (2.7)	28,718 (2.1)
Missing	238,610 (14.7)	32,923 (12.7)	205,687 (15.0)
**Social Vulnerability Index (SVI)**			
Mean (SD)	0.55 (0.26)	0.53 (0.25)	0.56 (0.26)
Quartile 1 (least vulnerable)	143,244 (8.8)	24,055 (9.3)	119,189 (8.7)
Quartile 2	499,480 (30.7)	89,576 (34.6)	409,904 (30.0)
Quartile 3	344,580 (21.2)	55,424 (21.4)	289,156 (21.1)
Quartile 4 (most vulnerable)	399,763 (24.6)	56,496 (21.9)	343,267 (25.1)
Unknown	238,790 (14.7)	32,972 (12.8)	205,818 (15.1)
**Clinical characteristics**			
**CCI score**			
0	813,575 (50.0)	82,304 (31.8)	731,271 (53.5)
1 to 3	565,258 (34.8)	108,886 (42.1)	456,372 (33.4)
≥4	229,069 (14.1)	67,333 (26.0)	161,736 (11.8)

© 2025 Vekaria V et al. *JAMA Network Open*.

Characteristic	Total, No. (%)	SMI, No. (%)	No SMI, No. (%)
Unknown	17,955 (1.1)	0 (0.0)	17,955 (1.3)
**Past-year healthcare services utilization**			
Mental health visit	457,601 (28.1)	199,972 (77.4)	257,629 (18.8)
Emergency department visit	100,687 (6.2)	49,008 (19.0)	51,679 (3.8)
Inpatient visit	73,028 (4.5)	38,692 (15.0)	34,336 (2.5)
Outpatient visit	401,356 (24.7)	181,827 (70.3)	219,529 (16.1)
Telehealth visit	115,851 (7.1)	60,071 (23.2)	55,780 (4.1)
**COVID-19 variant**			
Alpha	177,473 (10.9)	26,088 (10.1)	151,385 (11.1)
Ancestral	376,813 (23.2)	50,441 (19.5)	326,372 (23.9)
Delta	187,331 (11.5)	31,348 (12.1)	155,983 (11.4)
Omicron BA.1.1 - BA.2	380,862 (23.4)	62,925 (24.3)	317,937 (23.3)
Omicron BA.2.12.1 - BA.5	386,495 (23.8)	66,541 (25.7)	319,954 (23.4)
Omicron BQ.1.1 - XBB.1.5	116,883 (7.2)	21,180 (8.2)	95,703 (7.0)
**COVID-19 severity**			
Not hospitalized (least severe)	1,463,710 (90.0)	224,758 (86.9)	1,238,952 (90.6)
Incidental hospitalization	11,587 (0.7)	2,150 (0.8)	9,437 (0.7)
Hospitalized	114,873 (7.1)	25,758 (10.0)	89,115 (6.5)
Hospitalized with ventilation (most severe)	35,687 (2.2)	5,857 (2.3)	29,830 (2.2)
**COVID-19 treatment**			
Nirmatrelvir/ritonavir order	171,594 (10.6)	27,823 (10.8)	143,771 (10.5)
Remdesivir order	23,356 (1.4)	4,888 (1.9)	18,468 (1.4)
**COVID-19 death**	30,896 (1.9)	7,846 (3.0)	23,050 (1.7)
**PASC subtype**			
Blood-related	31,703 (1.9)	5,328 (2.1)	26,375 (1.9)
Circulatory-related	77,163 (4.7)	13,957 (5.4)	63,206 (4.6)
Digestive-related	74,039 (4.6)	11,807 (4.6)	62,232 (4.6)
Endocrine-related	76,476 (4.7)	12,857 (5.0)	63,619 (4.7)
Musculoskeletal-related	54,477 (3.4)	6,870 (2.7)	47,607 (3.5)
Neurological-related	133,203 (8.2)	26,470 (10.2)	106,733 (7.8)
Respiratory-related	107,434 (6.6)	16,603 (6.4)	90,831 (6.6)
Skin-related	16,932 (1.0)	3,647 (1.4)	13,285 (1.0)

© 2025 Vekaria V et al. *JAMA Network Open*.

eTable 5. Distribution of Patients With Social Vulnerability Indicators (First Quartile = Least Vulnerable, Fourth Quartile = Most Vulnerable)

		PASC status		Prior SMI status	
**Indicator**	**Total, No. (%)**	**No PASC, No. (%)**	**PASC, No. (%)**	**No SMI, No. (%)**	**SMI, No. (%)**
Total	1,625,857	1,222,216	403,641	1,367,334	258,523
**Socioeconomic status**					
Mean (SD)	0.50 (0.27)	0.49 (0.27)	0.51 (0.27)	0.50 (0.27)	0.48 (0.26)
Quartile 1	335,084 (20.6)	258,322 (21.1)	76,762 (19.0)	279,441 (20.4)	55,643 (21.5)
Quartile 2	390,182 (24.0)	297,555 (24.3)	92,627 (22.9)	320,419 (23.4)	69,763 (27.0)
Quartile 3	339,643 (20.9)	251,515 (20.6)	88,128 (21.8)	281,474 (20.6)	58,169 (22.5)
Quartile 4	322,158 (19.8)	238,691 (19.5)	83,467 (20.7)	280,182 (20.5)	41,976 (16.2)
Unknown	238,790 (14.7)	176,133 (14.4)	62,657 (15.5)	205,818 (15.1)	32,972 (12.8)
**Household characteristics**					
Mean (SD)	0.43 (0.27)	0.43 (0.27)	0.44 (0.27)	0.43 (0.27)	0.44 (0.26)
Quartile 1	445,130 (27.4)	337,836 (27.6)	107,294 (26.6)	378,077 (27.7)	67,053 (25.9)
Quartile 2	350,682 (21.6)	268,576 (22.0)	82,106 (20.3)	290,959 (21.3)	59,723 (23.1)
Quartile 3	368,351 (22.7)	272,212 (22.3)	96,139 (23.8)	302,099 (22.1)	66,252 (25.6)
Quartile 4	222,904 (13.7)	167,459 (13.7)	55,445 (13.7)	190,381 (13.9)	32,523 (12.6)
Unknown	238,790 (14.7)	176,133 (14.4)	62,657 (15.5)	205,818 (15.1)	32,972 (12.8)
**Racial/ethnic minority status**					
Mean (SD)	0.70 (0.22)	0.70 (0.22)	0.71 (0.22)	0.71 (0.22)	0.67 (0.22)
Quartile 1	64,653 (4.0)	48,495 (4.0)	16,158 (4.0)	51,915 (3.8)	12,738 (4.9)
Quartile 2	200,680 (12.3)	152,943 (12.5)	47,737 (11.8)	162,143 (11.9)	38,537 (14.9)
Quartile 3	456,194 (28.1)	352,373 (28.8)	103,821 (25.7)	371,933 (27.2)	84,261 (32.6)
Quartile 4	665,540 (40.9)	492,272 (40.3)	173,268 (42.9)	575,525 (42.1)	90,015 (34.8)
Unknown	238,790 (14.7)	176,133 (14.4)	62,657 (15.5)	205,818 (15.1)	32,972 (12.8)
**Housing/transport**					
Mean (SD)	0.63 (0.26)	0.63 (0.26)	0.64 (0.26)	0.63 (0.26)	0.61 (0.25)
Quartile 1	150,701 (9.3)	116,688 (9.5)	34,013 (8.4)	127,147 (9.3)	23,554 (9.1)
Quartile 2	209,370 (12.9)	155,760 (12.7)	53,610 (13.3)	173,392 (12.7)	35,978 (13.9)
Quartile 3	482,657 (29.7)	369,359 (30.2)	113,298 (28.1)	394,541 (28.9)	88,116 (34.1)
Quartile 4	544,339 (33.5)	404,276 (33.1)	140,063 (34.7)	466,436 (34.1)	77,903 (30.1)
Unknown	238,790 (14.7)	176,133 (14.4)	62,657 (15.5)	205,818 (15.1)	32,972 (12.8)

© 2025 Vekaria V et al. *JAMA Network Open*.

eTable 6. Logistic Regression Results Summarizing the Association Between Covariates and PASC Among Patients With SMI Only

	Unadjusted		Mutually adjusted	
**Predictor**	**OR (95% CI)**	***P* value**	**OR (95% CI)**	***P* value**
**Age at infection, y**				
22 to 34	1 [Reference]	—	1 [Reference]	—
35 to 44	1.03 (1.00–1.06)	.08	1.02 (0.99–1.05)	.20
45 to 64	1.12 (1.09–1.14)	<.001	1.07 (1.04–1.10)	<.001
≥65	1.26 (1.23–1.29)	<.001	1.11 (1.08–1.15)	<.001
**Sex**				
Female	1 [Reference]	—	1 [Reference]	—
Male	0.98 (0.96–0.99)	.011	0.93 (0.91–0.95)	<.001
**Race and ethnicity**				
Non-Hispanic White	1 [Reference]	—	1 [Reference]	—
Hispanic	1.11 (1.08–1.14)	<.001	1.08 (1.05–1.11)	<.001
Non-Hispanic Asian	1.02 (0.95–1.10)	.60	1.03 (0.95–1.10)	.50
Non-Hispanic Black	1.06 (1.03–1.09)	<.001	1.01 (0.98–1.04)	.50
Non-Hispanic other	1.10 (1.04–1.16)	<.001	1.06 (1.01–1.12)	.02
Missing or unknown	1.20 (1.17–1.23)	<.001	1.20 (1.17–1.23)	<.001
**Insurance type**				
Public	1 [Reference]	—	1 [Reference]	—
Commercial	0.83 (0.81–0.85)	<.001	0.88 (0.87–0.90)	<.001
Unknown or other	1.00 (0.97–1.02)	.70	1.00 (0.98–1.02)	>.90
**CCI score**				
0	1 [Reference]	—	1 [Reference]	—
1 to 3	1.10 (1.08–1.12)	<.001	1.03 (1.01–1.06)	.002
≥4	1.25 (1.22–1.28)	<.001	1.04 (1.01–1.06)	.006
Unknown				
**COVID-19 severity**	1 [Reference]	—	1 [Reference]	—
Not hospitalized (least severe)	1.48 (1.35–1.62)	<.001	1.45 (1.32–1.58)	<.001
Incidental hospitalization	1.75 (1.70–1.80)	<.001	1.68 (1.64–1.73)	<.001
Hospitalized	2.05 (1.95–2.16)	<.001	1.99 (1.89–2.10)	<.001

© 2025 Vekaria V et al. *JAMA Network Open*.

eTable 7. Logistic Regression Results Summarizing the Association Between Covariates and PASC Among Patients Without SMI Only

	**Unadjusted**		**Mutually adjusted**	
**Predictor**	**OR (95% CI)**	***P* value**	**OR (95% CI)**	***P* value**
**Age at infection, y**				
22 to 34	1 [Reference]	—	1 [Reference]	—
35 to 44	1.06 (1.05–1.08)	<.001	1.05 (1.04–1.06)	<.001
45 to 64	1.19 (1.17–1.20)	<.001	1.12 (1.11–1.14)	<.001
≥65	1.47 (1.45–1.49)	<.001	1.20 (1.18–1.21)	<.001
**Sex**				
Female	1 [Reference]	—	1 [Reference]	—
Male	0.92 (0.91–0.92)	<.001	0.87 (0.86–0.88)	<.001
**Race and ethnicity**				
Non-Hispanic White	1 [Reference]	—	1 [Reference]	—
Hispanic	1.13 (1.11–1.14)	<.001	1.13 (1.11–1.14)	<.001
Non-Hispanic Asian	0.96 (0.94–0.99)	.002	0.98 (0.96–1.00)	.08
Non-Hispanic Black	1.15 (1.14–1.17)	<.001	1.10 (1.09–1.12)	<.001
Non-Hispanic other	1.10 (1.07–1.12)	<.001	1.07 (1.05–1.09)	<.001
Missing or unknown	1.22 (1.21–1.24)	<.001	1.23 (1.21–1.24)	<.001
**Insurance type**				
Public	1 [Reference]	—	1 [Reference]	—
Commercial	0.73 (0.72–0.74)	<.001	0.84 (0.83–0.85)	<.001
Unknown or other	0.84 (0.83–0.85)	<.001	0.89 (0.88–0.90)	<.001
**CCI score**				
0	1 [Reference]	—	1 [Reference]	—
1 to 3	1.24 (1.23–1.25)	<.001	1.14 (1.13–1.15)	<.001
≥4	1.61 (1.59–1.62)	<.001	1.30 (1.28–1.32)	<.001
Unknown	0.76 (0.73–0.79)	<.001	0.73 (0.70–0.76)	<.001
**COVID-19 severity**				
Not hospitalized (least severe)	1 [Reference]	—	1 [Reference]	—
Incidental hospitalization	1.75 (1.67–1.82)	<.001	1.62 (1.55–1.69)	<.001
Hospitalized	2.04 (2.01–2.07)	<.001	1.83 (1.81–1.86)	<.001
Hospitalized with ventilation (most severe)	2.37 (2.31–2.42)	<.001	2.21 (2.15–2.26)	<.001

© 2025 Vekaria V et al. *JAMA Network Open*.

eTable 8. Logistic Regression Results Summarizing the Association Between Prior Schizophrenia and PASC

	Unadjusted		Mutually adjusted	
**Predictor**	**OR (95% CI)**	***P* value**	**OR (95% CI)**	***P* value**
Prior schizophrenia	1.31 (1.26–1.37)	<.001	1.07 (1.02–1.11)	.002
**Age at infection, y**				
22 to 34	1 [Reference]	—	1 [Reference]	—
35 to 44	1.06 (1.05–1.08)	<.001	1.05 (1.04–1.06)	<.001
45 to 64	1.19 (1.17–1.20)	<.001	1.12 (1.11–1.14)	<.001
≥65	1.47 (1.45–1.48)	<.001	1.20 (1.18–1.21)	<.001
**Sex**				
Female	1 [Reference]	—	1 [Reference]	—
Male	0.92 (0.91–0.92)	<.001	0.87 (0.87–0.88)	<.001
**Race and ethnicity**				
Non-Hispanic White	1 [Reference]	—	1 [Reference]	—
Hispanic	1.13 (1.11–1.14)	<.001	1.13 (1.11–1.14)	<.001
Non-Hispanic Asian	0.96 (0.94–0.98)	<.001	0.98 (0.96–1.00)	.06
Non-Hispanic Black	1.16 (1.14–1.17)	<.001	1.10 (1.09–1.12)	<.001
Non-Hispanic other	1.10 (1.07–1.12)	<.001	1.07 (1.05–1.09)	<.001
Missing or unknown	1.22 (1.21–1.23)	<.001	1.23 (1.21–1.24)	<.001
**Insurance Type**				
Public	1 [Reference]	—	1 [Reference]	—
Commercial	0.73 (0.72–0.74)	<.001	0.84 (0.83–0.85)	<.001
Unknown or other	0.84 (0.83–0.85)	<.001	0.89 (0.88–0.90)	<.001
**CCI score**				
0	1 [Reference]	—	1 [Reference]	—
1 to 3	1.24 (1.23–1.25)	<.001	1.14 (1.13–1.15)	<.001
≥4	1.60 (1.58–1.62)	<.001	1.29 (1.28–1.31)	<.001
Unknown	0.76 (0.73–0.79)	<.001	0.73 (0.70–0.76)	<.001
**COVID-19 severity**				
Not hospitalized (least severe)	1 [Reference]	—	1 [Reference]	—
Incidental hospitalization	1.74 (1.66–1.81)	<.001	1.61 (1.54–1.68)	<.001
Hospitalized	2.03 (2.00–2.06)	<.001	1.82 (1.80–1.85)	<.001
Hospitalized with ventilation (most severe)	2.35 (2.30–2.41)	<.001	2.19 (2.14–2.24)	<.001

© 2025 Vekaria V et al. *JAMA Network Open*.

eTable 9. Logistic Regression Results Summarizing the Association Between Prior Bipolar Disorder and PASC

	**Unadjusted**		**Mutually adjusted**	
**Predictor**	**OR (95% CI)**	***P* value**	**OR (95% CI)**	***P* value**
Prior bipolar disorder	1.27 (1.24–1.30)	<.001	1.14 (1.12–1.17)	<.001
**Age at infection, y**				
22 to 34	1 [Reference]	—	1 [Reference]	—
35 to 44	1.06 (1.05–1.08)	<.001	1.05 (1.04–1.06)	<.001
45 to 64	1.19 (1.17–1.20)	<.001	1.12 (1.11–1.14)	<.001
≥65	1.46 (1.44–1.48)	<.001	1.20 (1.18–1.21)	<.001
**Sex**				
Female	1 [Reference]	—	1 [Reference]	—
Male	0.91 (0.91–0.92)	<.001	0.87 (0.87–0.88)	<.001
**Race and ethnicity**				
Non-Hispanic White	1 [Reference]	—	1 [Reference]	—
Hispanic	1.12 (1.11–1.14)	<.001	1.12 (1.11–1.14)	<.001
Non-Hispanic Asian	0.96 (0.94–0.98)	<.001	0.98 (0.96–1.00)	.06
Non-Hispanic Black	1.15 (1.13–1.16)	<.001	1.10 (1.08–1.11)	<.001
Non-Hispanic other	1.09 (1.07–1.11)	<.001	1.07 (1.05–1.09)	<.001
Missing or unknown	1.22 (1.20–1.23)	<.001	1.23 (1.21–1.24)	<.001
**Insurance Type**				
Public	1 [Reference]	—	1 [Reference]	—
Commercial	0.73 (0.73–0.74)	<.001	0.84 (0.83–0.85)	<.001
Unknown or other	0.84 (0.83–0.85)	<.001	0.90 (0.89–0.91)	<.001
**CCI score**				
0	1 [Reference]	—	1 [Reference]	—
1 to 3	1.24 (1.23–1.25)	<.001	1.14 (1.13–1.15)	<.001
≥4	1.59 (1.57–1.61)	<.001	1.28 (1.27–1.30)	<.001
Unknown	0.76 (0.73–0.79)	<.001	0.73 (0.70–0.76)	<.001
**COVID-19 severity**				
Not hospitalized (least severe)	1 [Reference]	—	1 [Reference]	—
Incidental hospitalization	1.73 (1.66–1.80)	<.001	1.60 (1.54–1.67)	<.001
Hospitalized	2.03 (2.00–2.06)	<.001	1.82 (1.79–1.85)	<.001
Hospitalized with ventilation (most severe)	2.35 (2.29–2.40)	<.001	2.19 (2.14–2.24)	<.001

© 2025 Vekaria V et al. *JAMA Network Open*.

eTable 10. Logistic Regression Results Summarizing the Association Between Prior Recurrent MDD and PASC

	**Unadjusted**		**Mutually adjusted**	
**Predictor**	**OR (95% CI)**	***P* value**	**OR (95% CI)**	***P* value**
Prior recurrent MDD	1.18 (1.17–1.19)	<.001	1.08 (1.07–1.09)	<.001
**Age at infection, y**				
22 to 34	1 [Reference]	—	1 [Reference]	—
35 to 44	1.06 (1.05–1.07)	<.001	1.04 (1.03–1.06)	<.001
45 to 64	1.18 (1.17–1.19)	<.001	1.11 (1.10–1.12)	<.001
≥65	1.44 (1.42–1.45)	<.001	1.18 (1.17–1.20)	<.001
**Sex**				
Female	1 [Reference]	—	1 [Reference]	—
Male	0.91 (0.91–0.92)	<.001	0.88 (0.87–0.89)	<.001
**Race and ethnicity**				
Non-Hispanic White	1 [Reference]	—	1 [Reference]	—
Hispanic	1.11 (1.10–1.13)	<.001	1.12 (1.11–1.13)	<.001
Non-Hispanic Asian	0.95 (0.93–0.97)	<.001	0.98 (0.96–1.00)	.12
Non-Hispanic Black	1.13 (1.12–1.14)	<.001	1.08 (1.07–1.10)	<.001
Non-Hispanic other	1.08 (1.06–1.10)	<.001	1.07 (1.05–1.09)	<.001
Missing or unknown	1.21 (1.20–1.22)	<.001	1.22 (1.21–1.23)	<.001
**Insurance Type**				
Public	1 [Reference]	—	1 [Reference]	—
Commercial	0.74 (0.74–0.75)	<.001	0.85 (0.84–0.86)	<.001
Unknown or other	0.85 (0.84–0.86)	<.001	0.91 (0.90–0.92)	<.001
**CCI score**				
0	1 [Reference]	—	1 [Reference]	—
1 to 3	1.23 (1.22–1.24)	<.001	1.13 (1.12–1.14)	<.001
≥4	1.55 (1.53–1.57)	<.001	1.24 (1.22–1.25)	<.001
Unknown	0.75 (0.72–0.78)	<.001	0.72 (0.69–0.75)	<.001
**COVID-19 severity**				
Not hospitalized (least severe)	1 [Reference]	—	1 [Reference]	—
Incidental hospitalization	1.71 (1.64–1.77)	<.001	1.59 (1.53–1.66)	<.001
Hospitalized	2.00 (1.97–2.02)	<.001	1.80 (1.78–1.82)	<.001
Hospitalized with ventilation (most severe)	2.32 (2.27–2.37)	<.001	2.18 (2.13–2.23)	<.001

© 2025 Vekaria V et al. *JAMA Network Open*.

eTable 11. Logistic Regression Summary of Predictors of Developing PASC Including Adjustments for Social Vulnerability Indicators (Socioeconomic Status, Household Characteristics, Housing Type and Transportation)

	Unadjusted		Mutually adjusted	
**Predictor**	**OR (95% CI)**	***P* value**	**OR (95% CI)**	***P* value**
Prior Serious Mental Illness (SMI)	1.19 (1.18–1.20)	<.001	1.10 (1.09–1.11)	<.001
**Age at infection, y**				
22 to 34	1 [Reference]	—	1 [Reference]	—
35 to 44	1.06 (1.05–1.07)	<.001	1.06 (1.04–1.07)	<.001
45 to 64	1.18 (1.17–1.19)	<.001	1.13 (1.12–1.14)	<.001
≥65	1.44 (1.42–1.45)	<.001	1.21 (1.19–1.23)	<.001
**Sex**				
Female	1 [Reference]	—	1 [Reference]	—
Male	0.91 (0.91–0.92)	<.001	0.88 (0.87–0.89)	<.001
**Race and ethnicity**				
Non-Hispanic White	1 [Reference]	—	1 [Reference]	—
Hispanic	1.11 (1.10–1.12)	<.001	1.10 (1.09–1.12)	<.001
Non-Hispanic Asian	0.95 (0.93–0.97)	<.001	1.01 (0.99–1.04)	.30
Non-Hispanic Black	1.13 (1.12–1.14)	<.001	1.06 (1.05–1.07)	<.001
Non-Hispanic other	1.08 (1.06–1.10)	<.001	1.06 (1.04–1.09)	<.001
Missing or unknown	1.21 (1.19–1.22)	<.001	1.09 (1.08–1.11)	<.001
**Insurance Type**				
Public	1 [Reference]	—	1 [Reference]	—
Commercial	0.74 (0.74–0.75)	<.001	0.84 (0.83–0.85)	<.001
Unknown or other	0.85 (0.84–0.86)	<.001	0.92 (0.91–0.93)	<.001
**CCI score**				
0	1 [Reference]	—	1 [Reference]	—
1 to 3	1.23 (1.22–1.24)	<.001	1.13 (1.12–1.14)	<.001
≥4	1.55 (1.53–1.57)	<.001	1.22 (1.20–1.23)	<.001
Unknown	0.74 (0.72–0.77)	<.001	0.69 (0.66–0.72)	<.001
**COVID-19 severity**				
Not hospitalized (least severe)	1 [Reference]	—	1 [Reference]	—
Incidental hospitalization	1.70 (1.64–1.77)	<.001	1.69 (1.62–1.77)	<.001
Hospitalized	1.99 (1.97–2.02)	<.001	1.76 (1.74–1.79)	<.001
Hospitalized with ventilation (most severe)	2.31 (2.26–2.36)	<.001	2.05 (2.00–2.10)	<.001
**Socioeconomic status**				
Quartile 1 (least vulnerable)	1 [Reference]	—	1 [Reference]	—
Quartile 2	1.05 (1.04–1.06)	<.001	1.07 (1.05–1.08)	<.001
Quartile 3	1.18 (1.17–1.19)	<.001	1.13 (1.12–1.15)	<.001
Quartile 4 (most vulnerable)	1.18 (1.16–1.19)	<.001	1.13 (1.11–1.15)	<.001
**Household characteristics**				
Quartile 1 (least vulnerable)	1 [Reference]	—	1 [Reference]	—
Quartile 2	0.96 (0.95–0.97)	<.001	0.96 (0.95–0.97)	<.001
Quartile 3	1.11 (1.10–1.12)	<.001	1.02 (1.01–1.03)	<.001
Quartile 4 (most vulnerable)	1.04 (1.03–1.05)	<.001	0.93 (0.92–0.95)	<.001
**Housing type & transportation**				
Quartile 1 (least vulnerable)	1 [Reference]	—	1 [Reference]	—
Quartile 2	1.18 (1.16–1.20)	<.001	1.08 (1.06–1.10)	<.001
Quartile 3	1.05 (1.04–1.07)	<.001	0.97 (0.95–0.98)	<.001
Quartile 4 (most vulnerable)	1.19 (1.17–1.20)	<.001	1.00 (0.98–1.02)	.90

© 2025 Vekaria V et al. *JAMA Network Open*.
