# Supplementary material for: Schizophrenia, Bipolar, or Major Depressive Disorder and Postacute Sequelae of COVID-19
Source: JAMA Netw Open. 2025 Oct 29;8(10):e2540242. doi: 10.1001/jamanetworkopen.2025.40242 (PMC12573031; doi:10.1001/jamanetworkopen.2025.40242)
Supplement: Supplement 2. — Nonauthor Collaborators [file jamanetwopen-e2540242-s002.pdf]

*Group Name(s): RECOVER PCORnet EHR consortium							
*First Name and Middle Initial(s)	*Last Name	*Suffix (eg, Jr, III)	Academic Degrees	Institution	Location (city, state/province, country)	Role or Contribution, eg, chair, principal investigator	Group (if more than 1 Group listed in the byline) and/or Subgroup (eg, Steering Committee)
Selvin	Soby		Pharm.D	Albert Einstein College of Medicine	Bronx, NY, USA	Data Contributor	
Parsa	Mirhaji		Ph.D., M.D.	Albert Einstein College of Medicine	Bronx, NY, USA	Data Contributor	
Sara J.	Deakyne Davies		Ph.D., B.S.	Children's Hospital of Colorado	Aurora, CO, USA	Data Contributor	
Suchitra	Rao		M.D.	Children's Hospital of Colorado	Aurora, CO, USA	Data Contributor	
Priya	Alekapatti		M.S.	Cincinnati Children's	Cincinnati, OH, USA	Data Contributor	
Nathan M.	Pajor		M.S., M.D.	Cincinnati Children's	Cincinnati, OH, USA	Data Contributor	
Soumitra	Sengupta		B.E., M.S., Ph.D.	Columbia University	New York, NY, USA	Data Contributor	
Curtis	Kieler		B.S.E.	Duke University Health System	Durham, NC, USA	Data Contributor	
W. Schuyler	Jones		M.D.	Duke University Health System	Durham, NC, USA	Data Contributor	
Nita	Deshpande		M.D., B.A.	Emory University	Atlanta, GA, USA	Data Contributor	
Tony	Pan		ScB, Ph.D.	Emory University	Atlanta, GA, USA	Data Contributor	
Carol R.	Horowitz		M.D., MPH	Icahn School of Medicine at Mount Sinai	New York, NY, USA	Data Contributor	
Heidi T.	May		Ph.D., MSPH	InterMountain Healthcare	Salt Lake City, UT, USA	Data Contributor	
Benjamin D.	Horne		Ph.D., MStat, MPH, FAHA, FACC	InterMountain Healthcare	Salt Lake City, UT, USA	Data Contributor	
Bradley W.	Taylor		MBA, FAMIA	Medical College of Wisconsin	Milwaukee, WI, USA	Data Contributor	
Alexander	Stoddard		M.S.	Medical College of Wisconsin	Milwaukee, WI, USA	Data Contributor	
Reza	Shaker		M.D.	Medical College of Wisconsin	Milwaukee, WI, USA	Data Contributor	
David	Liebovitz		B.S., M.D.	Northwestern University	Evanston, IL, USA	Data Contributor	
Vesna	Mitrovic		Ph.D.	Northwestern University	Evanston, IL, USA	Data Contributor	
Saul	Blecker		M.D.	NYU Langone Health	New York, NY, USA	Data Contributor	
Nathalia	Ladino		B.S., M.S.	NYU Langone Health	New York, NY, USA	Data Contributor	
Marion R.	Sills		M.D., MPH	OCHIN	Portland, OR, USA	Data Contributor	
Soledad A.	Fernandez		Ph.D., MStat	Ohio State University	Columbus, OH, USA	Data Contributor	
Neena	Thomas		M.S., DipBA	Ohio State University	Columbus, OH, USA	Data Contributor	
Daniel	Fort		Ph.D., MPH, B.S.	Oschner Health	Jefferson, LA, USA	Data Contributor	
Wenke	Hwang		Ph.D.	Pennsylvania State College of Medicine	Hershey, PA, USA	Data Contributor	
Cynthia H.	Chuang		M.D., MSc	Pennsylvania State College of Medicine	Hershey, PA, USA	Data Contributor	
Alan	Schroeder		M.D.	Stanford University	Standford, CA, USA	Data Contributor	
Keith E.	Morse		M.D., MBA	Stanford University	Standford, CA, USA	Data Contributor	
Sharon J.	Herring		M.D., MPH	Temple University	Philadelphia, PA, USA	Data Contributor	
Yuriy	Bisyuk		M.D.	University Medical Center New Orleans (LSU	New Orleans, LA, USA	Data Contributor	
Mark J.	Pletcher		M.D., MPH	University of California San Francisco	San Francisco, CA, USA	Data Contributor	
Susan	Kim		M.D., MMSc	University of California San Francisco	San Francisco, CA, USA	Data Contributor	
Mei	Liu		Ph.D.	University of Florida	Gainesville, FL, USA	Data Contributor	

Jiang	Bian		M.S., Ph.D.	University of Florida	Gainesville, FL, USA	Data Contributor	
Elizabeth A.	Chrischilles		B.S., M.S., PharmD	University of Iowa	Iowa City, IA, USA	Data Contributor	
David A.	Williams		Ph.D.	University of Michigan	Ann Arbor, MI, USA	Data Contributor	
Xing	Song		Ph.D.	University of Missouri	Columbia, MO, USA	Data Contributor	
Abu S.	Mosa		B.S., M.S., Ph.D.	University of Missouri	Columbia, MO, USA	Data Contributor	
Jim	Svoboda		M.S.	University of Nebraska Medical Center	Omaha, NE, USA	Data Contributor	
Carol R.	Geary		Ph.D., MBA, RN	University of Nebraska Medical Center	Omaha, NE, USA	Data Contributor	
Michael J.	Becich		M.D., Ph.D.	University of Pittsburgh	Pittsburgh, PA, USA	Data Contributor	
Jonathan	Arnold		M.D., M.S.E.	University of Pittsburgh	Pittsburgh, PA, USA	Data Contributor	
Ramkiran	Gouripeddi		M.D., M.S.	University of Utah	Salt Lake City, UT, USA	Data Contributor	
Lindsay G.	Cowell		Ph.D.	UT Southwestern Medical Center	Dallas, TX, USA	Data Contributor	
Wei-Qi	Wei		M.D., Ph.D., FAMIA	Vanderbilt University Medical Center	Nashville, TN, USA	Data Contributor	
Rainu	Kaushal		M.D., M.S.	Weill Cornell Medicine	New York, NY, USA	Core Contributor	Data Contributor
Thomas	Campion		Ph.D., B.A., M.S.	Weill Cornell Medicine	New York, NY, USA	Core Contributor	Data Contributor
Thomas W.	Carton		Ph.D., M.S.	Louisiana Public Health Institute	New Orleans, LA, USA	Core Contributor	
Anna	Legrand		MPH	Louisiana Public Health Institute	New Orleans, LA, USA	Core Contributor	
Elizabeth	Nauman		MPH, Ph.D.	Louisiana Public Health Institute	New Orleans, LA, USA	Core Contributor	
Mark Weiner	Weiner		M.D., B.S.E.	Weill Cornell Medicine	New York, NY, USA	Core Contributor	
Sajjad Abedian	Abedian		M.S.	Weill Cornell Medicine	New York, NY, USA	Core Contributor	
Dominique	Brown		MPH	Weill Cornell Medicine	New York, NY, USA	Core Contributor	
Christopher	Cameron		Ph.D., B.A.	Weill Cornell Medicine	New York, NY, USA	Core Contributor	
Andrea	Cohen		M.P.A., M.S., M.B.A.	Weill Cornell Medicine	New York, NY, USA	Core Contributor	
Marietou	Dione		M.Sc, B.S.	Weill Cornell Medicine	New York, NY, USA	Core Contributor	
Rosie	Ferris		MPH	Weill Cornell Medicine	New York, NY, USA	Core Contributor	
Wilson	Jacobs		MPH	Weill Cornell Medicine	New York, NY, USA	Core Contributor	
Michael	Koropsak		M.S., B.S.	Weill Cornell Medicine	New York, NY, USA	Core Contributor	
Alexandra	LaMar		B.B.A	Weill Cornell Medicine	New York, NY, USA	Core Contributor	
Colby	Lewis	V	M.S.	Weill Cornell Medicine	New York, NY, USA	Core Contributor	
Dmitry	Morozyuk		B.S.	Weill Cornell Medicine	New York, NY, USA	Core Contributor	
Peter	Morrisey		B.S.	Weill Cornell Medicine	New York, NY, USA	Core Contributor	
Duncan	Orlander		M.S., B.A.	Weill Cornell Medicine	New York, NY, USA	Core Contributor	
Jyotishman	Pathak		Ph.D.	Weill Cornell Medicine	New York, NY, USA	Core Contributor	
Mahfuza	Sabiha		B.S.	Weill Cornell Medicine	New York, NY, USA	Core Contributor	
Edward J.	Schenck		M.D.	Weill Cornell Medicine	New York, NY, USA	Core Contributor	
Catherine	Sinfield		Ph.D., MPH	Weill Cornell Medicine	New York, NY, USA	Core Contributor	
Stephenson	Strobel		Ph.D.	Weill Cornell Medicine	New York, NY, USA	Core Contributor	
Zoe	Verzani		MPH	Weill Cornell Medicine	New York, NY, USA	Core Contributor	
Fei	Wang		Ph.D.	Weill Cornell Medicine	New York, NY, USA	Core Contributor	
Zhenxing	Xu		Ph.D.	Weill Cornell Medicine	New York, NY, USA	Core Contributor	
Chengxi	Zang		Ph.D.	Weill Cornell Medicine	New York, NY, USA	Core Contributor	
Yongkang	Zhang		Ph.D., M.S., B.B.A.	Weill Cornell Medicine	New York, NY, USA	Core Contributor	
