# Supplementary material for: Schizophrenia, Bipolar, or Major Depressive Disorder and Postacute Sequelae of COVID-19
Source: JAMA Netw Open. 2025 Oct 29;8(10):e2540242. doi: 10.1001/jamanetworkopen.2025.40242 (PMC12573031; doi:10.1001/jamanetworkopen.2025.40242)
Supplement: Supplement 3. — Data Sharing Statement [file jamanetwopen-e2540242-s003.pdf]

Data Sharing Statement

Vekaria. Schizophrenia, Bipolar, or Major Depressive Disorder With Postacute Sequelae of COVID-19. *JAMA Netw Open*. Published October 29, 2025.
doi:10.1001/jamanetworkopen.2025.40242

Data
Data available: No

Additional Information
Explanation for why data not available: Study uses a limited dataset of patient electronic health records that cannot be shared per data use agreement(s).
